# Impaired glucose metabolism and altered gut microbiome despite calorie restriction of ob/ob mice

**DOI:** 10.1186/s42523-019-0007-1

**Published:** 2019-09-05

**Authors:** Alireza Kashani, Asker Daniel Brejnrod, Chunyu Jin, Timo Kern, Andreas Nygaard Madsen, Louise Aas Holm, Georg K. Gerber, Jens-Christian Holm, Torben Hansen, Birgitte Holst, Manimozhiyan Arumugam

**Affiliations:** 1 Novo Nordisk Foundation Center for Basic Metabolic Research, Faculty of Health and Medical Sciences, University of Copenhagen, Blegdamsvej 3B, 2200 Copenhagen, Denmark; 2 Danish Diabetes Academy, Odense, Denmark; 3 Qbiom Microbiome Consultancy Service, Copenhagen, Denmark; 4 Department of Biomedical Sciences, Faculty of Health and Medical Sciences, University of Copenhagen, 2200 Copenhagen, Denmark; 5 The Children’s Obesity Clinic, Department of Paediatrics, Copenhagen University Hospital Holbæk, 4300 Holbæk, Denmark; 6 Department of Pathology, Brigham & Women’s Hospital, Harvard Medical School, Boston, MA USA; 7 Faculty of Health Sciences, University of Southern Denmark, Odense, Denmark

**Keywords:** Calorie restriction, Leptin deficiency, Leptin-deficient mice, Mouse gut microbiota, Obesity, Ob/Ob mice, 16S rRNA gene amplicon sequencing

## Abstract

**Background:**

Growing evidence supports the role of gut microbiota in obesity and its related disorders including type 2 diabetes. Ob/ob mice, which are hyperphagic due to leptin deficiency, are commonly used models of obesity and were instrumental in suggesting links between gut microbiota and obesity. Specific changes in their gut microbiota such as decreased microbial diversity and increased *Firmicutes* to *Bacteroidetes* ratio have been suggested to contribute to obesity via increased microbiota capacity to harvest energy. However, the differential development of ob/ob mouse gut microbiota compared to wild type microbiota and the role of hyperphagia in their metabolic impairment have not been investigated thoroughly.

**Results:**

We performed a 10-week long study in ob/ob (*n* = 12) and wild type control (*n* = 12) mice fed ad libitum. To differentiate effects of leptin deficiency from hyperphagia, we pair-fed an additional group of ob/ob mice (*n* = 11) based on the food consumption of control mice. Compared to control mice, ob/ob mice fed ad libitum exhibited compromised glucose metabolism and increased body fat percentage. Pair-fed ob/ob mice exhibited even more compromised glucose metabolism and maintained strikingly similar high body fat percentage at the cost of lean body mass. Acclimatization of the microbiota to our facility took up to 5 weeks. Leptin deficiency impacted gut microbial composition, explaining 18.3% of the variance. Pair-feeding also altered several taxa, although the overall community composition at the end of the study was not significantly different. We found 24 microbial taxa associations with leptin deficiency, notably enrichment of members of *Lactobacillus* and depletion of *Akkermansia muciniphila*. Microbial metabolic functions related to energy harvest, including glycan degradation, phosphotransferase systems and ABC transporters, were enriched in the ob/ob mice. Taxa previously reported as relevant for obesity were associated with body weight, including *Oscillibacter* and *Alistipes* (both negatively correlated) and *Prevotella* (positively correlated).

**Conclusions:**

Leptin deficiency caused major changes in the mouse gut microbiota composition. Several microbial taxa were associated with body composition. Pair-fed mice maintained a pre-set high proportion of body fat despite reduced calorie intake, and exhibited more compromised glucose metabolism, with major implications for treatment options for genetically obese individuals.

**Electronic supplementary material:**

The online version of this article (10.1186/s42523-019-0007-1) contains supplementary material, which is available to authorized users.

## Background

Gut microbiota, the trillions of microbes living in the gut, can be thought of as a functional metabolic organ that complements the host’s metabolic apparatus. They have been associated with many diseases in humans, including obesity [1], type 2 diabetes [2, 3] and other metabolic diseases. Recent studies have shown that diet can rapidly and reproducibly alter the human gut microbiota [4] and that transplanting the gut microbiota from human donors into germ-free mice can reproducibly transmit donor body composition phenotypes such as adiposity [5, 6]. Fecal transplants in humans have also suggested that microbes can improve metabolic health [7]. These results indicate a potential for manipulating the gut microbial composition to achieve a desired phenotype, such as decreasing adiposity and ameliorating metabolic disorders, and improve human health.

Studies on the leptin-deficient (ob/ob) mouse model for obesity have shown that, compared to normal wild type mice, ob/ob mice have markedly different gut microbial composition and increased capacity to harvest energy from food [8]. Colonization of germ-free wild type mice with ob/ob mouse microbiota led to a significantly greater increase of adiposity compared to colonization from lean mouse microbiota [8], hinting that this increased capacity may be transmissible. These results suggest that there is a bidirectional host-microbial cross-talk where the host selects the microbiota depending on its genotype/phenotype (e.g., leptin deficiency), and the microbiota influences the host phenotype (e.g., adiposity). However, studies so far have not described the longitudinal development of gut microbiota of ob/ob mice. Thus, we still do not know how the host and the gut microbiota shape each other over time in ob/ob mice, and how this process differs from wild type mice.

We conducted a longitudinal study on ob/ob mice (OB) and wild type controls (WT) fed ad libitum. We studied the differences in gut microbiota between ob/ob and wild type mice in order to understand the effect of leptin deficiency on the gut microbiota. To further investigate whether the differences are due to leptin deficiency or hyperphagia, we studied a third group of ob/ob mice that was pair-fed based on the food consumption of the wild type mice (OB-PF). We characterized the development of their body composition as well as their gut microbiota, and investigated how these two diverged over time between these three groups. This setup enabled us to identify microbial markers associated with genetically induced obesity as well as restricted calorie intake that is commonly used to mitigate obesity.

## Results

We obtained mice that were 5 weeks old from a commercial vendor. We used 16S ribosomal RNA gene amplicon sequencing (targeting the V4 variable region) to characterize their fecal microbiota over 9 weeks (W1-W9) and cecal microbiota in week 11 (Additional file 4: Table S1). We generated 2.8 million high quality paired-end reads from 348 samples (average 7866 and minimum 5795 paired-end reads per sample), and bioinformatically analyzed them using the DADA2 package [9]. Altogether, the three groups of mice – wild type fed ad libitum (WT), ob/ob fed ad libitum (OB), ob/ob pair-fed based on consumption of WT mice (OB-PF) – harbored a total of 1136 amplicon sequence variants [10] (ASVs), each potentially representing an individual microbial strain.

### Pair-feeding-induced calorie restriction compromises metabolism in ob/ob mice

**Fig. 1:**
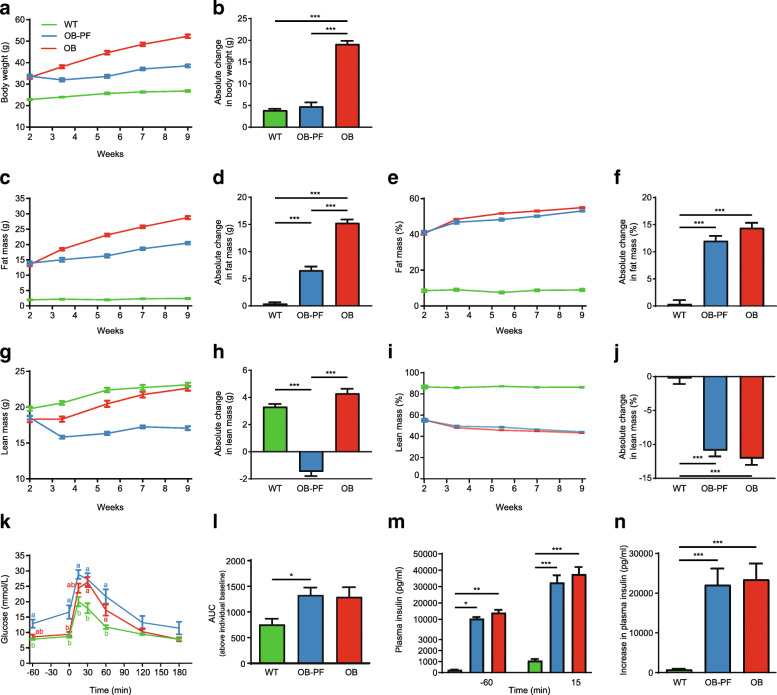
Body composition monitored from week 2, approximately every second week for 7 weeks. **a-b** Body weight development (**a**) and absolute weight gain (**b**). **c-d** Fat mass in grams (**c**) and absolute change in fat mass (**d**). **e-f** Fat mass as percentage of body weight (**e**) and absolute change in fat mass percentage (**f**). **g-h** Lean body mass in grams (**g**) and absolute change in lean body mass (**h**). **i-j** Lean mass as percentage of body weight (**i**) and absolute change in lean mass percentage (**j**). **k** Absolute blood glucose levels before and after oral glucose administration during oral glucose tolerance test conducted in week 10. Both OB-PF-vs-OB and OB-PF-vs-WT comparisons have statistically significant differences (*P* < 0.01) following one-way ANOVA. Statistical significance from multiple comparison test following two-way repeated measures ANOVA are shown using letter-based codes: within each time point, pairs of groups that do not share a letter show statistically significant (*P* < 0.05) difference. See Additional file 4: Table S9 for all comparisons. **l** Area under the curve above baseline blood glucose of individual groups. **m-n** Plasma insulin concentration 60 min before and 15 min after glucose was administered orally (**m**) and increase in plasma insulin (**n**). Comparisons with statistically significant differences are denoted with significance levels as: ***P < 0.001, **P < 0.01, *P < 0.05

During the course of the study, both WT and OB mice gradually gained body weight while feeding ad libitum on normal chow (Fig. 1a). OB mice gained significantly higher body weight over a 7-week period compared to WT mice (*P* < 0.001; Fig. 1b). However, OB-PF mice lost body weight immediately after the start of pair-feeding (Fig. 1a). They regained body weight afterwards and exhibited a net gain in body weight comparable to WT mice over the study period (Fig. 1b). OB mice continuously gained fat mass throughout the study (Fig. 1c). In OB-PF mice, pair-feeding did not seem to eliminate fat accumulation, as they also continuously gained fat mass during the same period (Fig. 1c), although significantly less compared to OB mice as indicated by the net gain over the study (*P* < 0.001; Fig. 1d). WT mice gained the lowest fat mass (Fig. 1d) and maintained the highest absolute lean mass among the three groups (Fig. 1g). Interestingly, while both WT and OB mice gradually gained lean body mass, OB-PF mice lost on average 14.7% of their lean mass in the first 10 days of calorie-restriction (Fig. 1g). Despite a slow recovery in the following weeks, OB-PF mice had a net loss in lean body mass over the study period, while OB and WT mice had a net gain in lean body mass (Fig. 1h). Compared to WT mice, OB and OB-PF mice maintained nearly identical higher fat mass percentage (Fig. 1e) and nearly identical lower lean mass percentage (Fig. 1i). Over the study period, the net gain of fat mass percentage and net loss of lean mass percentage of OB and OB-PF groups were significantly higher than that of WT mice (*P* < 0.001; Fig. 1f, j).

In week 10, we performed an oral glucose tolerance test (OGTT) to compare the glucose metabolism of the groups. Prior to the OGTT after 12-h fasting, OB-PF mice showed significantly increased fasting blood glucose levels compared to both OB and WT mice (*P* < 0.01 and *P* < 0.001, respectively; Fig. 1k, 0 min; see Additional file 4: Table S9). As expected, both OB and OB-PF groups showed impaired glucose concentration curves during OGTT. While the impaired glucose tolerance of the OB-PF mice was more pronounced than that of OB mice, the difference between OB-PF and OB was not statistically significant when considering the AUC above individual baseline fasting glucose levels (Fig. 1l). This suggests that the perceived increase was primarily due to the increased fasting glucose concentrations in OB-PF mice, rather than an impaired insulin response. Both OB and OB-PF mice had significantly higher fasting plasma insulin concentrations compared to WT mice (*P* < 0.01 and *P* < 0.05, respectively; Fig. 1m, − 60 min). Fifteen minutes after glucose administration, they also had a significant increase in plasma insulin compared to the WT mice (both *P* < 0.001; Fig. 1n). However, we did not observe any significant difference between OB and OB-PF mice in either fasting insulin concentrations or their insulin secretion in response to glucose challenge (Fig. 1m, n), confirming that OB-PF mice had comparable insulin resistance to OB mice, which may reflect the similar fat percentage. OB-PF mice did exhibit a significantly worse glucose metabolism compared to WT (Fig. 1l, n).

### Leptin deficiency, calorie restriction and environment affect the gut microbial composition

**Fig. 2:**
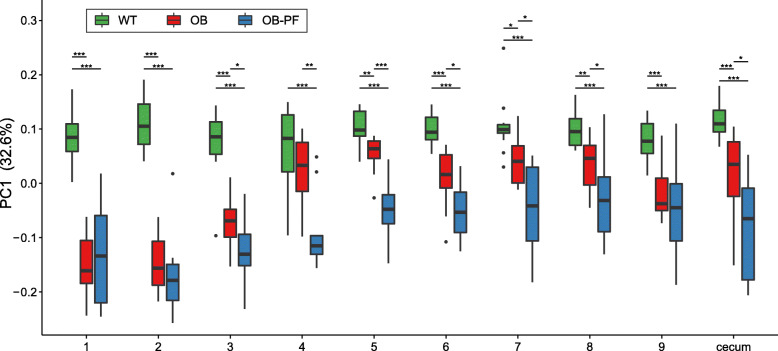
Separation of gut microbial composition in the three groups. First principle coordinate shows separation of wild-type mice fed ad libitum (WT), ob/ob mice fed ad libitum (OB) and ob/ob mice pair-fed according to WT intake (OB-PF). Principal coordinate analysis was performed on fecal samples from weeks 1–9 and cecum sampled at week 11, using Jensen-Shannon Divergence as the beta-diversity measure. Comparisons with statistically significant differences (see Additional file 4: Table S4) are denoted with significance levels as: ***P < 0.001, **P < 0.01, *P < 0.05

We performed Principal Coordinate Analysis [11] using Jensen-Shannon Divergence (JSD) to investigate the variation of the microbial composition over time. JSD is derived from relative entropy and measures the dissimilarity between given two probability distributions [12]. Figure 2 illustrates the variation of the first principal coordinate (PC1) over time, and also between the WT, OB and OB-PF mice. Even though a few samples were identified as outliers in some weeks, no mouse was consistently marked as an outlier across weeks. PC1 of the WT mice was significantly different from the OB and OB-PF mice during almost all time points (8 and 9, respectively, out of 9 time points; Wilcoxon rank-sum test, *P* < 0.05; Additional file 4: Table S4). Based on PC1, we observed that during W3-W5 fecal microbial communities of both OB and OB-PF mice gradually became more similar to the WT mice (Fig. 2). This was likely due to the acclimatization of the mice to the new environment in our animal housing facility. This was then followed by the stronger influence of leptin deficiency and pair-feeding during W6-W9. As expected, OB and OB-PF mice did not show any significant differences between them on arrival at our animal facility and until 2 weeks of pair-feeding (Wilcoxon rank-sum test, *P* = 0.898 and *P* = 0.303 during W1 and W2, respectively). They gradually diverged from each other starting in W3 (*P* = 0.032) and showing significant differences in W4 and W5 (*P* = 0.005 and *P* = 0.0007, respectively). During W6-W8, they still maintained differences albeit at a lower statistical significance (0.020 < *P* < 0.032). During almost all weeks, both OB and OB-PF mice had a significantly different PC1 score compared to WT mice (Fig. 2). While both OB and OB-PF mice diverged from the WT mice in similar directions of microbial community composition, divergence of OB-PF showed higher magnitude. This suggests that starvation experienced by the OB-PF mice or the severe decrease in lean mass had a strong impact in shaping the composition of the microbiota.

**Fig. 3:**
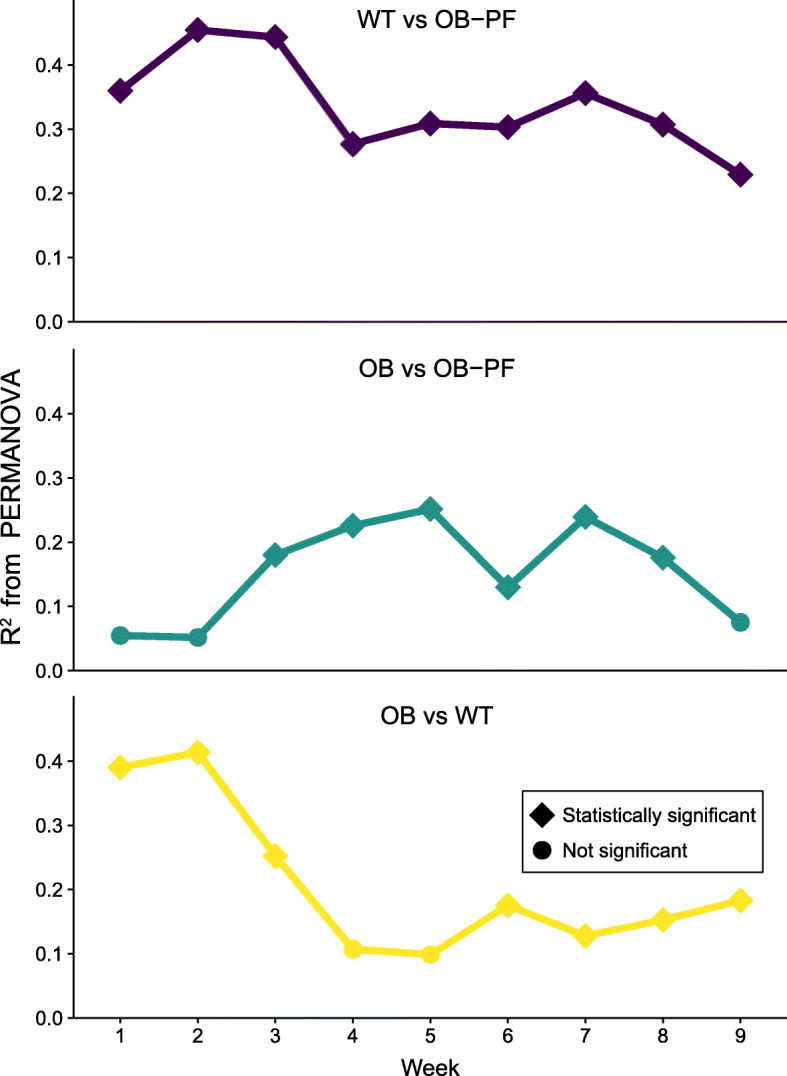
Separation of microbial profiles of mice in the three groups: wild-type mice fed ad libitum (WT), ob/ob mice fed ad libitum (OB) and ob/ob mice pair-fed according to WT intake (OB-PF). PERMANOVA tests were performed for the different time points comparing pairs of the three groups. For each pair of groups, variance (R^2^) explained by the group membership estimated by PERMANOVA test is shown. Shapes denote whether the groups were significantly different during the comparison

We then investigated how the primary factors involved in our study design – leptin deficiency and pair-feeding – individually affected the gut microbial composition. We used the permutational multivariate analysis of variance (PERMANOVA) test on microbial community dissimilarities (in this case JSD) estimated in time-stratified pairwise comparisons. We used the variance explained (R^2^) in the PERMANOVA test as an indication of differences in microbial composition as illustrated in Fig. 3, with R^2^ = 0 being no variance explained by the group difference, and R^2^ = 1 meaning all variance explained by the group difference. During the first week, differences in microbial composition were the biggest in the OB-vs-WT and WT-vs-OB-PF comparisons across genotypes. As expected, prior to pair-feeding, comparison of OB-PF and OB groups delivered from the same vendor with identical genotype and feeding regimen did not show statistically significant differences (R^2^ = 5.5%; *P* = 0.319). Just after acclimatization in W6, the feeding regimen separated the OB-PF from OB (R^2^ = 13.0%, *P* = 0.002), but its effect reduced by W9 showing only a separation trend (R^2^ = 7.5%, *P* = 0.11), suggesting that the microbiota was accommodating to the substantial physiological impact of the feeding regimen. The across-genotype comparisons at W9 (OB-vs-WT and WT-vs-OB-PF) both showed substantial separation (R^2^ = 18.3% and R^2^ = 22.9%, respectively, *P* = 0.001), suggesting that the ob/ob genotype had a larger effect on microbial composition.

At termination of the experiment on W11, samples were taken from cecal content. We compared the fecal microbiota from W9 to the cecal microbiota at W11. The difference between compartments explained only 3.2% of the variance in the microbiome, while the group (WT, OB or OB-PF) explained 22.2% of the variance (*P* = 0.01 and *P* = 0.001, respectively). This suggests that the difference between the microbiome composition of the three groups was significantly larger than the difference between fecal and cecal microbiota.

### Lack of consistent associations between gut microbial alpha diversity and obesity

We looked for associations between individual body weight and microbial alpha diversity measures using linear mixed models. Both ASV richness and Shannon diversity were significantly negatively associated with body weight (*P* = 0.036 and *P* = 0.0024, respectively; Additional file 4: Table S2), suggesting that gut microbial diversity may be associated with individual body weight.

**Fig. 4:**
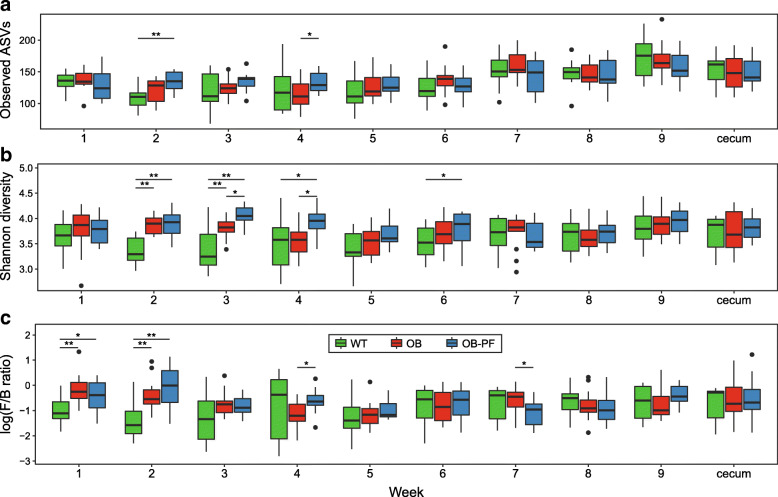
Overall characteristics of the gut microbiomes sampled from weeks 1–9 and cecum sampled at week 11. **a**-**b** Gut microbial alpha diversity of wild-type mice fed ad libitum (WT), ob/ob mice fed ad libitum (OB) and ob/ob mice pair-fed according to WT intake (OB-PF). Two different measures are shown: observed ASV richness (**a**) and Shannon diversity (**b**). **c** *Firmicutes* to *Bacteroidetes* ratios in gut microbiota of WT, OB and OB-PF mice, shown in log-scale. Comparisons with statistically significant differences are denoted with significance levels as: ***P < 0.001, **P < 0.01, *P < 0.05

We compared ASV richness and Shannon diversity between the groups over time. Neither the fecal microbiota nor the cecal microbiota showed any significant differences in ASV richness between WT and OB mice (Fig. 4a; Wilcoxon rank-sum test, *P* > 0.23; see Additional file 4: Table S3). However, OB-PF mice exhibited significantly higher richness compared to OB in W4 (Wilcoxon rank-sum test, *P* = 0.024). During W2 and W3, both OB and OB-PF mice showed significantly higher fecal microbial diversity than WT mice, measured by Shannon index (Fig. 4b; Wilcoxon rank-sum test, *P* < 0.005). OB-PF mice also had higher Shannon index compared to WT in W4 and W6; and also had higher Shannon index compared to OB in W3 and W4 (Wilcoxon rank-sum test, *P* < 0.05).

We then compared *Firmicutes* to *Bacteroidetes* (F/B) ratios of cecal and fecal microbiota between the WT and OB mice. F/B ratio of cecal microbiota was not significantly different between the two groups (Fig. 4c; Wilcoxon rank-sum test, *P* = 0.55). When comparing their fecal microbiota, OB mice exhibited a significantly higher F/B ratio compared to WT mice only during W1 and W2 (Wilcoxon rank-sum test, *P* < 0.005, see Additional file 4: Table S3), and borderline significant higher F/B ratio during W3 (*P* = 0.064). We did not observe any significant difference between WT and OB mice during other weeks (Wilcoxon rank-sum test, *P* > 0.35).

### Consistent gut microbial features associated with leptin deficiency

**Fig. 5:**
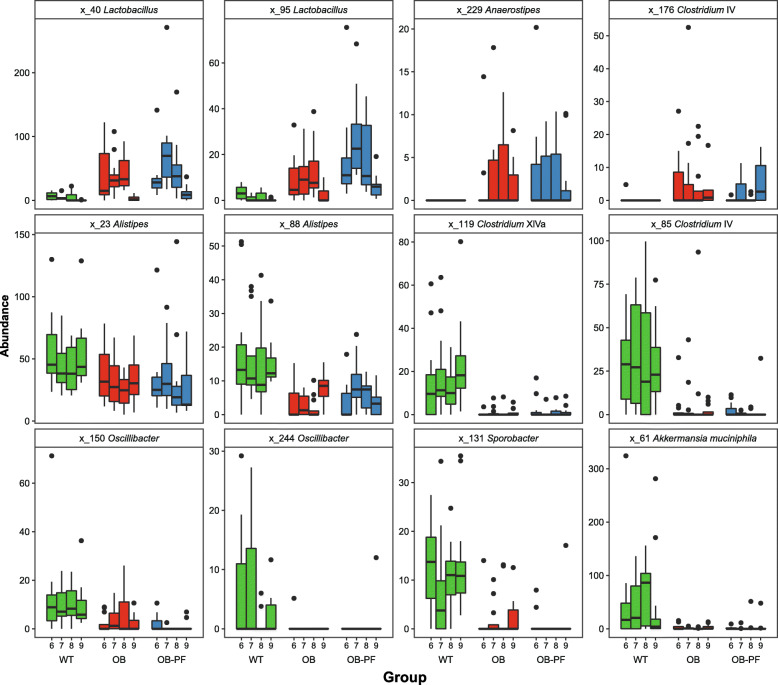
Microbial taxa that are differentially abundant in ob/ob mice. In total, 28 ASVs were consistently differentially abundant during weeks 6–9 in both OB and OB-PF mice compared to WT mice. Twelve ASVs annotated at least to the genus level are shown here. Library size normalized read counts are shown for each week within each group. Four ASVs in the first row are enriched in ob/ob mice, and eight ASVs in second and third rows are depleted in ob/ob mice

We searched for differentially abundant ASVs between the groups using pairwise comparisons. We used the negative binomial Wald test for pairwise comparisons during each week, and identified several ASVs that were differentially abundant (Additional file 1: Figure S1 and Additional file 4: Table S8). Number of differentially abundant ASVs between WT and OB groups decreased from 50 in W2 to 6 in W3. We did not find any differentially abundant ASVs between WT and OB groups during W4–W5, which agrees with the convergence of OB group towards WT group in Fig. 2. In order to eliminate the acclimatization process as a confounding factor, we compared samples only from W6 to W9, while controlling for age using Wilcoxon rank-sum test. We identified 43, 77 and 28 ASVs that were differentially abundant in WT-vs-OB, WT-vs-OB-PF and OB-vs-OB-PF comparisons, respectively (*P* < 0.05; Additional file 4: Table S5). To investigate changes associated with the ob/ob genotype regardless of pair-feeding, we identified taxa that were consistently differentially abundant in both OB and OB-PF groups compared to WT group. We observed that 10 ASVs were enriched in the ob/ob phenotype and 18 ASVs were depleted. Two ASVs from *Lactobacillus*, one ASV from *Anaerostipes* and one ASV from *Clostridium* cluster IV were notable among those enriched in ob/ob phenotype (Fig. 5). Two ASVs from *Alistipes*, two ASVs from *Oscillibacter*, one ASV each from Clostridium clusters XIVa and IV, and an ASV from *Akkermansia muciniphila* were notable among those depleted in ob/ob phenotype (Fig. 5).

We predicted the microbial functional potential profiles from the taxonomic abundance profiles using Piphillin [13]. We identified 14, 11 and 2 KEGG pathways that were differentially abundant in WT-vs-OB, WT-vs-OB-PF and OB-vs-OB-PF comparisons, respectively; and 39, 72 and 8 KEGG orthologous groups that were differentially abundant in WT-vs-OB, WT-vs-OB-PF and OB-vs-OB-PF comparisons, respectively (*P* < 0.05; Wilcoxon rank-sum test controlled for age; Additional file 4: Table S6). Similar to taxonomic analysis, we looked for consistent differential abundance of functional potential in both OB and OB-PF mice compared to WT mice. In ob/ob mice, we observed an enrichment of three KEGG pathways and 29 KEGG orthologous groups, together with a depletion of one pathway (Glycosphingolipid biosynthesis - ganglio series) and five orthologous groups (*P* < 0.05; Wilcoxon rank-sum test controlled for age; see Additional file 4: Table S6 for full list). We observed the enrichment of the phosphotransferase system (PTS) pathway, glutathione metabolism pathway, 7 orthologous groups corresponding to ABC transporters including 3 polar amino acid transport systems, 5 orthologous groups corresponding to PTS system and 2 orthologous groups corresponding to glycan degradation.

### Temporal modelling of microbial networks reveals differential topology

**Fig. 6:**
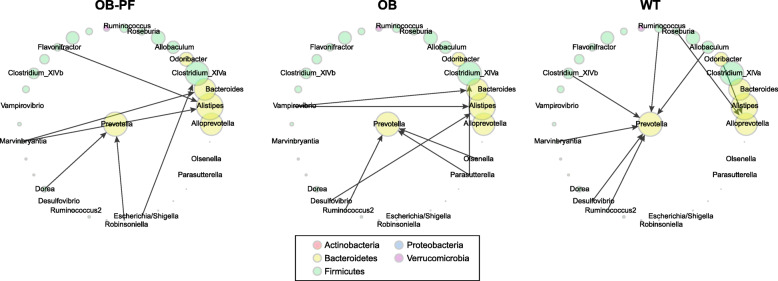
Granger-causality networks at genus level. Granger-causality networks were made for the three groups: wild-type mice fed ad libitum (WT), ob/ob mice fed ad libitum (OB) and ob/ob mice pair-fed according to WT intake (OB-PF). Node size reflects the relative abundance of the genus, and node color denotes the phylum

We elucidated the community structure of the microbiome by incorporating temporal information via the concept of Granger-causality. Despite the name, Granger-causality does not establish causality in the classical sense, but in the rather narrow sense that one member of the community can predict another, making a stronger suggestion that they might be causally linked rather than mere correlation. In particular, Granger-causality is not well-suited for unobserved variables that might drive the variation in both the predicted and the independent members. Thus, we performed this analysis only within treatment groups using genus level abundances, to observe the behaviour of the microbiota within the major driver of variation. This resulted in a directed network suggesting which genera can predict other genera (Fig. 6). OB-PF, OB and WT groups had 6, 7 and 8 connections, respectively. We observed that *Prevotella* was an important node in all networks. Its abundance could be predicted by several low-abundance Gram-positive genera. WT mice also had *Alloprevotella* predicted by *Roseburia*. Pearson correlation between *Alloprevotella* abundances and time-lagged *Roseburia* abundances revealed that the association was negative (PCC = − 0.86, *P* = 0.006). OB and OB-PF mice had many connections between less abundant clades and highly abundant *Bacteroidetes* genera such as *Prevotella*, *Bacteroides* and *Alistipes*. In OB mice, the time-lagged abundances of *Vampirovibrio* were strongly positively correlated with *Alistipes* (PCC = 0.93, *P* = 0.0006). This pattern was disrupted in the OB-PF group, where other genera were predictive of *Alistipes*.

### Gut microbial members associated with host body composition and metabolic phenotype

We looked for associations between members of the gut microbiota and host metabolic parameters. Since the three groups of mice exhibited different metabolic states, we performed the analysis for each group independently. First, for OGTT-AUC and 2-h insulin concentrations estimated only once at W10, we estimated the Spearman correlation coefficient between ASVs and the phenotype of interest. We only considered associations with absolute Spearman’s ρ above 0.4 and adjusted *p*-value below 0.05 (Additional file 4: Table S7a ,b). One ASV from *Ruminococcaceae* family negatively correlated with OGTT-AUC only in the OB group (ρ = − 0.9). Three *Bacteroidales* ASVs correlated with 2-h insulin concentrations, also only in the OB group – one ASV each from *Bacteroides acidifaciens* and *Alistipes* correlated positively, while an ASV from *Muribaculaceae* family correlated negatively.

We then investigated correlations of taxa with the various body composition measurements collected over 10 weeks – body weight, lean body mass and fat mass (all in grams). Age was highly correlated with these measurements (body weight: ρ > 0.74 in all groups; lean body mass: ρ > 0.63 in WT and OB groups; fat mass: ρ > 0.81 in OB and OB-PF groups). Therefore, we constructed linear mixed models to adjust for the effect of age and the individual mice (Additional file 2: Figure S2; see Additional file 4: Table S7c-e for associated ASVs). We found that 21 ASVs were associated with overall body weight in OB group, 8 ASVs were associated in OB-PF group, but none in WT group (Additional file 4: Table S7c). Among these, *Muribaculaceae*, *Lachnospiraceae* and *Ruminococcaceae* families had multiple ASVs associated. We found 9 ASVs to be significantly associated with lean body mass in the WT group (all negatively) and 13 ASVs in the OB-PF group (12 negatively). Finally, 3 ASVs were negatively associated with fat mass in the OB group.

## Discussion

We performed a longitudinal study investigating the development of gut microbiota in three groups of mice: wild type controls fed ad libitum (WT), leptin-deficient ob/ob mice fed ad libitum (OB), and ob/ob mice fed on par with WT controls (OB-PF). We found that the gut microbiota underwent drastic changes over a 5-week long process, where the ob/ob (both OB and OB-PF) and WT gut microbiota converged towards each other. We hypothesize that this trend was the effect of the acclimatization process in a new environment in our animal facility influencing the gut microbiota of the three groups to become more similar during W1-W5, as has been shown previously [14]. Contrary to a recent study suggesting a shorter acclimatization period of 7 days [15], our results suggest that this period could be significantly longer.

Leptin deficiency leads to constant hunger, which leads to hyperphagia and obesity. We clearly see this trend in our OB mice fed ad libitum. These mice also continuously gained fat mass – while WT mice maintained an almost constant fat mass percentage around 10%, OB mice increased their fat percentage from 40% in W1 to 55% in W8. These findings agree with a previous report [16]. These results suggest that leptin deficiency reprograms the mice to preserve a pre-set high proportion of body fat. It appears to be irrespective of calorie intake, as OB-PF mice achieved this high fat proportion, despite consuming only 50–70% of the calories as OB mice (Additional file 3: Figure S3). They appear to achieve this also by losing lean mass when necessary, thereby having significantly lower absolute lean mass compared to OB mice, thus exhibiting a net loss in lean body mass over the study period. This suggests that the body likely defends fat mass over lean body mass, where insulin resistance could be instrumental in preserving fat mass (that normally leads to obesity). Based on the OGTT glucose concentration curves, OB-PF mice had worse glucose metabolism than OB mice, who themselves were worse than WT. However, after adjusting for individual baseline blood glucose values, only OB-PF mice had significantly higher AUC from the OGTT curves compared to WT mice. OB-PF mice had elevated fasting glucose concentrations, yet showed a significantly higher AUC compared to WT mice even after adjusting for individual baseline glucose values. This could be an important mechanism behind the OB-PF mice regaining fat mass. Both the OGTT curve and longitudinal trend in the lean body mass suggest that OB-PF mice unexpectedly had a higher magnitude of compromised glucose metabolism compared to OB mice. This needs to be considered for treatment of leptin-deficient obese humans, as simply reducing calories to that of a healthy person’s intake may not reduce the body fat percentage, and might even worsen metabolism. Our results also raise an interesting question whether lean mass decline during starvation could be a proxy for fat mass preservation ability, thereby helping us choose appropriate therapy for obese individuals.

Summary metrics of the microbiome, such as the F/B ratio and alpha diversity, have received a lot of attention in the literature. Many of these observations are cross-sectional, i.e. a single observation collected from a group of humans or mice. This has led to both conflicting reports within host species and doubts about translation across host species. Evaluating cecal microbiota of ob/ob mice and lean littermates, Ley et al. reported that F/B ratio was lower in obese mice than in lean mice [17]. This was later confirmed and elaborated that the altered microbiota had an increased capacity to harvest energy [8]. Later studies reported conflicting results about associations between obesity and alpha-diversity, both in mice [16] and in humans [18, 19].

Here we systematically evaluated gut microbial alpha diversity and F/B ratio as markers of obesity in mice in a longitudinal setup and found inconsistent trends over time. We observed several timepoints at which statistically significant differences between groups were present, but there was no strong omnipresent trend for any group. Obviously, cross-sectional sampling at these points would have led to conclusions that might not be observed in the preceding or following weeks. This suggests that the inconclusive body of literature so far might be the result of observing a static picture of the dynamic ecological priority effects intertwined with the inherent residual confounding occurring when a group of free-living humans is chosen for a study based on a complex phenotype such as obesity.

OB mice had 43 differentially abundant ASVs compared to WT mice, suggesting that this phenotype could be exerting fat mass preserving strategies at least partially through the microbiota. However, OB-PF mice had 77 differentially abundant ASVs compared to WT mice, and 28 such ASVs compared to OB mice. OB-PF mice were under stress, starving, and potentially influenced by a biological mechanism to preserve fat mass, which could be driving these additional changes in their gut microbiota. We observed that 28 ASVs were differentially abundant in both OB and OB-PF groups compared to WT. Leptin plays an important role in immune responses and inflammation, and is suggested to be part of the cytokine cascade that is behind host defense mechanisms [20]. This could explain the high number of differentially abundant ASVs in the gut microbiota of the ob/ob mice.

We observed an enrichment of two ASVs under genus *Lactobacillus* in ob/ob mice, which is surprising as *Lactobacillus* spp. are considered beneficial. While some studies have reported positive association between *Lactobacillus* spp. and obesity [21, 22], there are others that showed beneficial effects of probiotic supplementation with *Lactobacillus* on obesity [23]. Even systematic reviews performed by different authors came to contradictory conclusions on the effect of *Lactobacillus* supplementation on body weight and BMI [24, 25]. As suggested previously, the role of *Lactobacillus* spp. in obesity needs to be investigated further in better-designed studies [23].

One of the five *Lachnospiraceae* ASVs enriched in ob/ob mice was a member of *Anaerostipes* genus. A previous study demonstrated that strain AJ110941 closely related to *Anaerostipes* contributed to metabolic dysfunction of ob/ob mice [26]. One of the five *Lachnospiraceae* ASVs depleted in ob/ob mice belonged to *Clostridium* cluster XIVa, which are major butyrate producers in gut [27], supporting a link between inflammation and obesity. Cani et al showed that members of this cluster were decreased in high-fat-diet-fed obese mice that also showed increased metabolic endotoxemia [28].

We also observed a depletion of *Akkermansia muciniphila* in the ob/ob mice. *A. muciniphila* is a mucin-degrading Gram-negative bacterium, which resides in the mucus layer. Abundance of this species has been widely reported to associate negatively with body fat and glucose intolerance in mice and in humans [29–31]. Administration of *A. muciniphila* prevented the development of obesity and associated complications, leading to correction of metabolic endotoxemia in obese and diabetic mice [31], suggesting that the depletion of *A. muciniphila* in ob/ob mice could be more than just an association.

At the microbiome functional level, enrichment of PTS enzymes in both OB and OB-PF mice is in accordance with previous reports [32]. PTS is found only in bacteria where it catalyzes the transport and phosphorylation of numerous monosaccharides, disaccharides, amino sugars, polyols, and other sugar derivatives into the bacterial cell. Our results strengthen the previous hypothesis of increased energy harvest in microbiome of the ob/ob mice [33]. PTS enzymes have also been reported to be enriched in human fecal microbiota of obese and inflammatory bowel diseases patients [34]. A previous study had demonstrated that high-fat carbohydrate diet in humanized gnotobiotic mice correlates with an enhanced proportion of PTS and ABC transporters [18]. While there was no significant difference in PTS enzymes between OB and OB-PF mice, the enrichment level compared to WT mice was higher in OB-PF mice than in OB mice. This additional increase in PTS could also be linked to an increased ability of microbes to assimilate all available sugars to compensate for the reduced dietary intake in OB-PF mice, as proposed previously [35]. This observation is unique to our study design that could evaluate the energy harvest capacity of ob/ob mice under starvation.

Microbial networks have emerged as an important tool for investigating the ecology of microbiomes. While many networks have been generated in cross-sectional settings [3, 36], longitudinal sampling can add additional information about dynamics that otherwise cannot be observed [37]. Granger-causality networks [38] use longitudinal information to explore possible causal connections in the microbiota. Our networks suggest different ecological interactions in the different groups, suggesting that genotype alone may not drive such interactions. There are many possible ways bacteria could causally influence abundance such as competition, cross-feeding of nutrients, quorum sensing, changing of micro-environments and active killing via secondary metabolites. For example, active killing could be a venue of further investigation for the interactions between *Roseburia* and *Alloprevotella*, as members of the *Roseburia* clade have been known to produce several bacteriocins [39, 40], and the abundance of *Roseburia* in a given week was negatively associated with *Alloprevotella* abundance in the following week. Similarly, the OB group has the curious case of *Vampirovibrio*, which is low-abundant but seemingly predictive of several Gram-negative clades. Some members of *Vampirovibrio* are known to be predators of other bacteria, presumably using type VI secretion systems [41], and interestingly have previously been isolated from human gut [42]. Such predatory activities might underlie the negative effect of *Vampirovibrio* on other bacteria.

Longitudinal associations via correlations of glucose metabolism, insulin secretion, body weight, lean body mass and fat mass suggested many taxa that could be related to body composition. Interestingly, all 14 ASVs from *Firmicutes* associated with body weight in the OB group showed only negative correlations. *Prevotella,* positively correlated with body weight in the OB group, has been associated with insulin resistance in humans [43]. *Alistipes*, negatively correlated with both fat mass and body weight in the OB group, has previously been shown to be enriched in human obesity and type 2 diabetes [44]. More importantly, it was also shown to be enriched in people who succeeded in weight loss intervention [45], agreeing with the negative correlation found in our study. Further studies are needed to characterize whether the associated microbial taxa found in our study indeed contribute to changes in the body composition of mice.

## Conclusions

Our results demonstrate that leptin-deficient ob/ob mice have a unique gut microbial composition that progressively and consistently differs from the wild type mice over time, with severe changes in the microbial network topology. Several microbial taxa were associated with body composition, suggesting a host-microbial cross-talk in metabolism. We highlight that despite a reduced calorie intake on par with wild type mice, pair-fed ob/ob mice maintained a high body fat percentage, which occurred concomitantly with a net loss in lean body mass and an impaired glucose metabolism. Future research into treatment options for genetically obese patients should take these into consideration.

## Methods

### Mice and experimental design

#### Description of mice

Ob/ob mice and lean control mice (ob/+ and +/+ littermates of ob/ob mice) with C57BL/6 J genetic background were purchased from Janvier Labs. They were maintained on a 12-h light/12-h dark cycle with lights off at 6 pm and individually housed throughout the study. The animals were 5 weeks old when the study started.

#### Study design

We conducted a longitudinal study on the ob/ob and lean wild type control mice with C57BL/6 J genetic background. Lean wild type control mice were fed ad libitum (referred to as WT, *n* = 12). Half of the ob/ob mice were fed ad libitum (referred to as OB, *n* = 12) whereas the other half were fed based on the average food intake in the WT group. We refer to this group as the pair-fed group (OB-PF, *n* = 11). The average daily food intake per mouse in the WT group was calculated based on the weekly consumption and the same amount of food was subsequently given to OB-PF mice. The food intake data are shown in Additional file 3: Fig. S3. Due to the capacity of equipment, the mice were handled in two batches, where batch B entered the experiment 1 week later than batch A. Each batch consisted of all 3 groups with 5–6 animals each. To characterize the development of the gut microbiota and investigate microbial changes over time among groups, fresh fecal samples were collected once per week for nine consecutive weeks. Cecum content was harvested at the end of the study when the animals were euthanized. Body composition and weight of the animals were measured approximately every second week, starting from week 2. OGTT was conducted in week 10. In week 11, the animals were euthanized and the cecum content was collected.

#### Diet composition

The diet used in this study was Altromin 1319 - Extrude (Altromin Spezialfutter GmbH, Germany) which is a cereal-based formula and designed as complete feed for rats and mice. Its metabolized energy is ~ 3339 kcal/kg, 14% from fat, 27% from protein and 59% from carbohydrates. Its content of crude nutrients and moisture is 11.1% moisture, 6.1% crude ash, 4.5% crude fibre, 5.1% crude fat, 22.5% crude protein and 50.7% Nitrogen-free extractives.

#### Measurements during the study

Body composition: Body composition of the mice including fat and lean mass was determined by quantitative magnetic resonance imaging (MRI) using EchoMRI (Echo Medical Systems, Houston, TX, USA).

OGTT: Mice were fasted for 12 h from 9 pm with free access to water. Glucose (1.5 g/kg body weight) was administered orally. Concentrations of blood glucose were measured with a handheld glucometer (Ascensia Contour Glucometer, Bayer) using blood obtained from the punctures on the tail along a time course before and after glucose load. At time points of -60 and 15 min, blood samples were collected from the orbital sinus for insulin analysis (Sensitive Insulin RIA kit, Linco Research).

#### Collection of fresh feces and cecum content

Once per week, freshly defecated feces were directly collected into eppendorf tubes and frozen immediately in liquid nitrogen. At the end of the study, the whole intestinal tract was dissected. The cecum was cut off and then cut open. The content was scooped out, transferred into an eppendorf tube and immediately frozen in liquid nitrogen. All the samples were stored at -80 °C until further analysis.

### Microbiome analysis

#### DNA extraction, 16S rRNA library preparation and sequencing

Genomic DNA was isolated from 200 mg of fecal samples using the NucleoSpin Soil kit (Macherey-Nagel GmbH & Co. KG, Germany) following manufacturer’s instructions. SL2 + Enhancer buffer SX were used as the cell lysis buffer, the subsequent vortex step was replaced with repeated bead beating. DNA yield, purity and integrity were assessed using a Qubit 2.0 fluorometer, a NanoDrop 2000 spectrometer (Thermo Fisher Scientific Inc., MA USA) and agarose gel electrophoresis, respectively. Library preparation with polymerase chain reaction (PCR) amplification was performed using 20 ng bacterial DNA, 0.2 μM of each barcoded forward and reverse primer, and HotMasterMix (5 Prime) solution in a total volume of 25 μl. To target the variable region 4 (V4) of the 16S rRNA gene, a forward primer 515F (5′ AATGATACGGCGACCACCGAGATCTACAC<i5>TATGGTAATTGTGTGCCAGCMGCCGCGGTAA 3′) and a reverse primer 806R (5′ AAGCAGAAGACGGCATACGAGAT<i7>AGTCAGTCAGCCGGACTACHVGGGTWTCTAAT 3′) were used; each primer consisted the appropriate Illumina adapter, an 8-nt index sequence i5 and i7, a 10-nt pad sequence, a 2-nt linker, and the gene-specific primer [46, 47]. The PCR reaction conditions were 3 min at 94 °C, followed by 28 cycles of 20 s at 94 °C, 30 s at 55 °C and 54 s at 72 °C on an Eppendorf thermocycler (Eppendorf AG, Germany). Amplicons were purified with a magnetic-bead based clean-up and size selection kit (Macherey-Nagel GmbH & Co. KG, Germany). Amplicons were visualized by gel electrophoresis and quantified by a Qubit 2.0 fluorometer. A master DNA pool was generated from the purified products in equimolar ratios. The DNA was sequenced using an Illumina MiSeq platform (MiSeq Reagent Kits v2, 500 cycles), generating a total of 14,657,899 paired-end reads. After removing 2 samples with insufficient sequencing depth (<100 paired-end reads each), we obtained 348 samples varying from 9327 to 114,923 with median 38,930 paired-end reads per sample.

#### Sequence analysis and building taxonomic abundance table

We used the high-resolution DADA2 method [9] to infer the exact sequences from amplicons. Unlike OTUs, this does not impose any arbitrary threshold and thereby obtains inferred amplicon sequence variants (ASVs) that differ by as little as one nucleotide [10].

Based on the FastQC [48] plots of sequence reads, we chose to truncate the forward and reverse reads at position 240 and 150 respectively. In addition, we trimmed the first 10 nucleotides of each read (trimLeft = 10) based on empirical observation across our previously sequenced samples as well as recommendation by DADA2 developers [49]. We discarded reads with any ambiguous nucleotides (maxN = 0) as well as filtered out reads with more than two expected errors (maxEE = 2). We de-replicated the final sequences in order to remove redundancy. We finally generated 7.3 million high quality paired-end reads from 348 samples (average 21010 and minimum 5795 paired-end reads per sample).

DADA2 can correct sequencing errors through a probabilistic noise model for wrong base calls by incorporating both the quality scores and sequence frequencies. We estimated the error rates using 180 out of 348 samples. We used the per-sample inference mode (pool = FALSE). We removed chimeric sequences using *removeBimeraDenovo* function. We used *assignSpecies* in combination with *RDP species-level training set version 14* database provided with DADA2 to assign taxonomy to ASVs. Finally a phyloseq (v 1.19.1) object [50] was generated within the R environment to proceed for the analysis. We excluded ASVs with unknown phylum assignment, which could be artifacts. This resulted in 1136 ASVs.

#### Resolving ambiguous taxonomic assignments in Porphyromonadaceae family

A recent study described a novel family in *Bacteroidetes* phylum, which is dominant in mouse gut microbiota, and proposed that it should be named *Muribaculaceae* [51]. Currently, this novel family is part of *Porphyromonadaceae* family in RDP database but resolved separately as *Bacteroidales* S24–7 family in SILVA database. Our taxonomic annotation procedure using RDP classified all ASVs from this novel family as *Porphyromonadaceae* spp. However, the newly reported [51] phylogeny of clades related to *Porphyromonadaceae* family is more accurate than the RDP phylogeny we used. Therefore, to better resolve taxonomic assignments of these taxa, we re-annotated 102 ASVs originally assigned by RDP to *Porphyromonadaceae* family using the database from the integrated Mouse Gut Metagenomic Catalog [52] (https://github.com/tillrobin/iMGMC, commit 53d5583). Among them, 20 could not be annotated by this database and we thus kept the original taxonomic annotation from RDP. Of the rest, 72 were reclassified as *Bacteroidales* S24–7 group at family level, 2 assigned to *Bacteroidales* at order level, and only 8 remained in *Porphyromonadaceae* family. ASVs with this altered taxonomy are marked in Additional file 4: Tables S5 and S7. Throughout the manuscript, we refer to *Bacteroidales* S24–7 family as *Muribaculaceae* to be consistent with the newly proposed nomenclature.

To investigate if there were batch effects, for each time point we compared the two batches within each group (WT, OB, and OB-PF) using two methods of differential abundance (Wilcoxon ranksum test and DESeq2) and did not find any ASVs that were differentially abundant.

#### Predicting microbial functional potential

We predicted the microbial functional potential profiles from the taxonomic abundance profiles using Piphillin [13]. As input, we used ASVs after excluding taxa with average relative abundance less than 0.01% across samples, and prevalence of < 10% all 9 weeks of the study. Inference was run with default parameters at the Piphillin web resource (https://piphillin.secondgenome.com). Profiles were obtained at KEGG orthologous group and pathway levels.

### Statistical analyses

#### Statistical analysis of animal body composition and glucose metabolism data

Data are presented as mean ± SE and were analyzed using statistical software GraphPad Prism 7 (GraphPad, San Diego, CA, USA). One-way ANOVA or two-way repeated measures ANOVA followed by Tukey’s multiple comparisons test was carried out. Results were considered significant when *P <* 0.05.

#### Rarefaction and library size normalization

For fair estimations of alpha-diversity (ASV richness and Shannon diversity), samples were rarefied to 5795 reads. Rarefaction was not performed for *Firmicutes* to *Bacteroidetes* ratio, as this ratio will not be affected by sequencing depth. These rarefied estimates were used in (i) groupwise comparisons of alpha-diversity and (ii) associations between alpha-diversity and phenotypes.

For accurate estimation ASV abundances across samples, we used DESeq2 (v 1.14.1) for normalizing our read count data of all 1136 ASVs to adjust for the sequencing library size [53]. These normalized counts were used for (i) ASV differential abundance tests using DESeq2 for estimating batch effects, (ii) overall ASV differential abundance tests using Wilcoxon rank-sum tests consistent across W6-W9, (iii) ASV differential abundance tests using DESeq2 within each time point and (iv) association between ASV abundances and phenotypes. When sequencing depth is above ~ 1000 sequences per sample (which is the case for our study with minimum 5795 sequences per sample), rarefaction is not more effective than other normalization techniques, but library size must be accounted for [54]. Additionally, in differential abundance analysis, rarefaction leads to lower power because data has been thrown away [54, 55]. Therefore, we preferred DESeq2 library size normalization rather than rarefaction. Since DESeq2 library size normalization does not affect relative abundance data, differential abundance tests using Wilcoxon rank-sum test and beta diversity analysis using ASV relative abundance (see below) are unaffected by this library size normalization.

#### Alpha diversity and Firmicutes to Bacteroidetes (F/B) ratio

Alpha diversity was computed using phyloseq’s plot_richness() function after rarefying the data to 5795 reads per sample. We used Wilcoxon test implemented in the coin (v 1.2.2) package [56] to assess differences between groups in terms of ASV richness, Shannon diversity and F/B ratio within each week.

#### Beta diversity

We filtered out taxa with average relative abundance less than 1% across samples. We estimated beta diversity using Jensen-Shannon divergence (JSD) as implemented in phyloseq [50]. PERMANOVA analysis was performed on the resulting distance table using *adonis2* function implemented in the vegan package [57] with 999 permutations. First principal coordinate (PC1) based on JSD was compared between groups within each week.

#### Differential abundance tests

For differential abundance analysis, we excluded taxa with average relative abundance less than 0.01% across samples, and prevalence of < 10% all time points (9 weeks of fecal samples and cecal samples at termination). This resulted in 337 ASVs.

For samples from a given week, we used the negative binomial Wald test implemented in DESeq2 for differential abundance analysis of pairs of groups. Taxa with adjusted *p*-value less than 0.05 (as estimated by DESeq2) were considered statistically significant.

We used the Wilcoxon test implementation in the coin (v 1.2.2) package with week number (time) as blocking factor to identify taxa that were consistently significantly different during W6-W9. DESeq2 cannot perform such an analysis by modeling the effect of time. The fold change was calculated as the average over medians of variation of taxa in a given group across time, over the same quantity in another group. All reported *p*-values are adjusted for multiple hypothesis testing using Holm’s sequential rejection procedure. Taxa with adjusted *p*-value less than 0.05 were considered statistically significant.

We also used Wilcoxon test implementation in the coin (v 1.2.2) package with week number (time) as blocking factor to identify functions (KEGG pathways and KEGG orthologous groups) that were significantly different during W6-W9. We excluded pathways or orthologous groups with average relative abundance less than 0.1% across samples. All reported *p*-values are adjusted for multiple hypothesis testing using Holm’s sequential rejection procedure. Functions with adjusted *p*-value less than 0.05 were considered statistically significant.

#### Assessing significant association of alpha diversity with metabolic phenotype

Alpha diversity was computed using phyloseq’s plot_richness() function after rarefying the data to 5795 reads per sample. Models were fitted with the lme4 R package [58], using the phenotypes (body weight, lean mass, and fat mass) as outcomes and the alpha diversity as predictor, adjusted for group and time. Effect sizes (betas) and FDR-adjusted *p*-values are reported in Additional file 4: Table S2.

#### Assessing significant association of ASV relative abundances with metabolic phenotype

To model the outcomes body weight, lean body mass and fat mass using microbial features, linear mixed models were fitted using the lme4 package [58] within each group, for every ASV with mean relative abundance higher than 0.01% within that group. Z-scored (standard scores obtained using mean and standard deviation of all observations of an ASV) relative ASV abundances and time were used as fixed effects, while animal identifiers were modeled as a random intercept. *P*-values for ASV beta coefficients were obtained through the lmerTest package [59] using the Satterthwaite method. For OGTT-AUC and 2-h insulin concentrations estimated only once at W10, we estimated the Spearman correlation coefficient between ASVs and the phenotype of interest using cor.test() function, within each group. *P*-values were corrected within each outcome using FDR.

#### Granger-causality network

Granger-causal networks were built in Matlab 2013b with the Lasso-granger package [38]. Averaged genus trajectories within groups were used as predictors. We predicted based on a lag of 1, and genera were selected with a lambda of 1. Genera included had a summed relative abundance > 10^− 3^. Networks were plotted with igraph [60].

## Additional files

Additional file 1: **Figure S1.** Volcano plots separated by weeks showing gut microbial taxa that are differentially abundant in pairwise comparisons of wild-type mice fed ad libitum (WT), ob/ob mice fed ad libitum (OB) and ob/ob mice pair-fed according to WT intake (OB-PF). X-axis shows fold changes (in log2 scale) and Y-axis shows unadjusted *p*-values as reported by DESeq2 (in negative log10 scale). Each point represents an ASV, and its color represents whether a given ASV is significantly differentially abundant in that comparison after adjusting for multiple correction (as reported by DESeq2). (PDF 5436 kb)

Additional file 2: **Figure S2.** Heatmaps showing associations between ASVs and host metabolic phenotypes within the three groups: wild-type mice fed ad libitum (WT), ob/ob mice fed ad libitum (OB) and ob/ob mice pair-fed according to WT intake (OB-PF). Associations that are not statistically significant are shown with white cells. (A) Effect size from linear mixed models between ASVs and three phenotypes: body weight, lean body mass and fat mass. (B) Spearman correlation co-efficient between ASVs and two phenotypes: area-under-the-curve for glucose response curves and insulin levels 2 h after glucose administration. (PDF 256 kb)

Additional file 3: **Figure S3.** Daily food intake of wild-type mice fed ad libitum (WT), ob/ob mice fed ad libitum (OB) and ob/ob mice pair-fed according to WT intake (OB-PF). Intake was precisely measured with TSE system (PhenoMaster, Bad Homburg, Germany) in week 3 (A) and week 9 (B). ***P < 0.001, for WT-vs-OB and OB-PF-vs-OB comparisons. (PDF 47 kb)

Additional file 4: **Table S1.** Detailed information on when fresh fecal samples and cecum content were collected. **Table S2.** Associations between individual metabolic phenotypes and microbial alpha diversity measures using linear mixed models. **Table S3.** Testing for differences in the Shannon diversity index, observed ASVs, and Firmicutes to Bacteroidetes ratio between the groups during each week. FDR-adjusted p-values from Wilcoxon rank-sum test are reported. **Table S4.** Testing for differences in PC1 between groups during each week. FDR-adjusted p-values from Wilcoxon rank-sum test are reported. **Table S5a-c.** ASVs that are differentially abundant in pairwise comparisons of groups, consistently during W6-W9. Both p-values and Holm's sequential rejection-based adjusted p-values are reported. ASVs re-annotated with iMGMC are marked in the last column "Altered taxonomy". **Table S6a-f.** KEGG pathways (**a-c**) and orthologous groups (**d-f**) that are differentially abundant in pairwise comparisons of groups, consistently during W6-W9. Both p-values and Holm's sequential rejection-based adjusted p-values are reported. **Table S7a-b.** Spearman rank correlations between microbial taxa and one-time metabolic measurements, estimated separately for the groups. Taxa with at least one correlation with FDR-adjusted *P* < 0.05 or absolute value of Spearman's rho over 0.4 are reported. **Table S7c-e.** Correlations between microbial taxa and body weight (**c**), lean body mass (**d**) and fat mass (**e**) measured multiple times. Linear mixed models were estimated separately for the groups. Taxa with at least one correlation with FDR-adjusted *P* < 0.05 are reported. **Table S8a-c.** ASVs that are differentially abundant in pairwise comparisons of groups during each week, tested using DESeq2. Both unadjusted and adjusted p-values from DESeq2 are reported. **Table S9.** Tukey's multiple comparison test results for pairwise comparisons of blood glucose levels during an oral glucose tolerance test in the three groups. (XLSX 174 kb)

## Data Availability

Sequencing reads have been deposited at NCBI Short Read Archive under BioProject identifier PRJNA400789. Additional file data representing mouse phenotypes, processed microbiome data including relative abundances of taxonomic/functional features and microbiome pairwise distances can be downloaded at http://arumugamlab.sund.ku.dk/SuppData/Kashani_et_al_2019_OBOB/.

## Abbreviations

ABC transporters: ATP-binding cassette transporters
ASV: Amplicon sequence variant
AUC: Area under the curve
F/B ratio: *Firmicutes* to *Bacteroidetes* ratio
JSD: Jensen-Shannon Divergence
KEGG: Kyoto Encyclopedia of Genes and Genomes
OB: Ob/ob mice fed ad libitum
OB-PF: Ob/ob mice pair-fed based on food intake of wild type control mice
OGTT: Oral glucose tolerance test
PCC: Pearson correlation coefficient
PERMANOVA: Permutational multivariate analysis of variance
PTS: Phosphotransferase system
W1, W2: Week 1, week 2
WT: Wild type control mice fed ad libitum
