# Supplementary figures and images for: Impaired glucose metabolism and altered gut microbiome despite calorie restriction of ob/ob mice
Source: Anim Microbiome. 2019 Sep 5;1:11. doi: 10.1186/s42523-019-0007-1 (PMC7807779; doi:10.1186/s42523-019-0007-1)

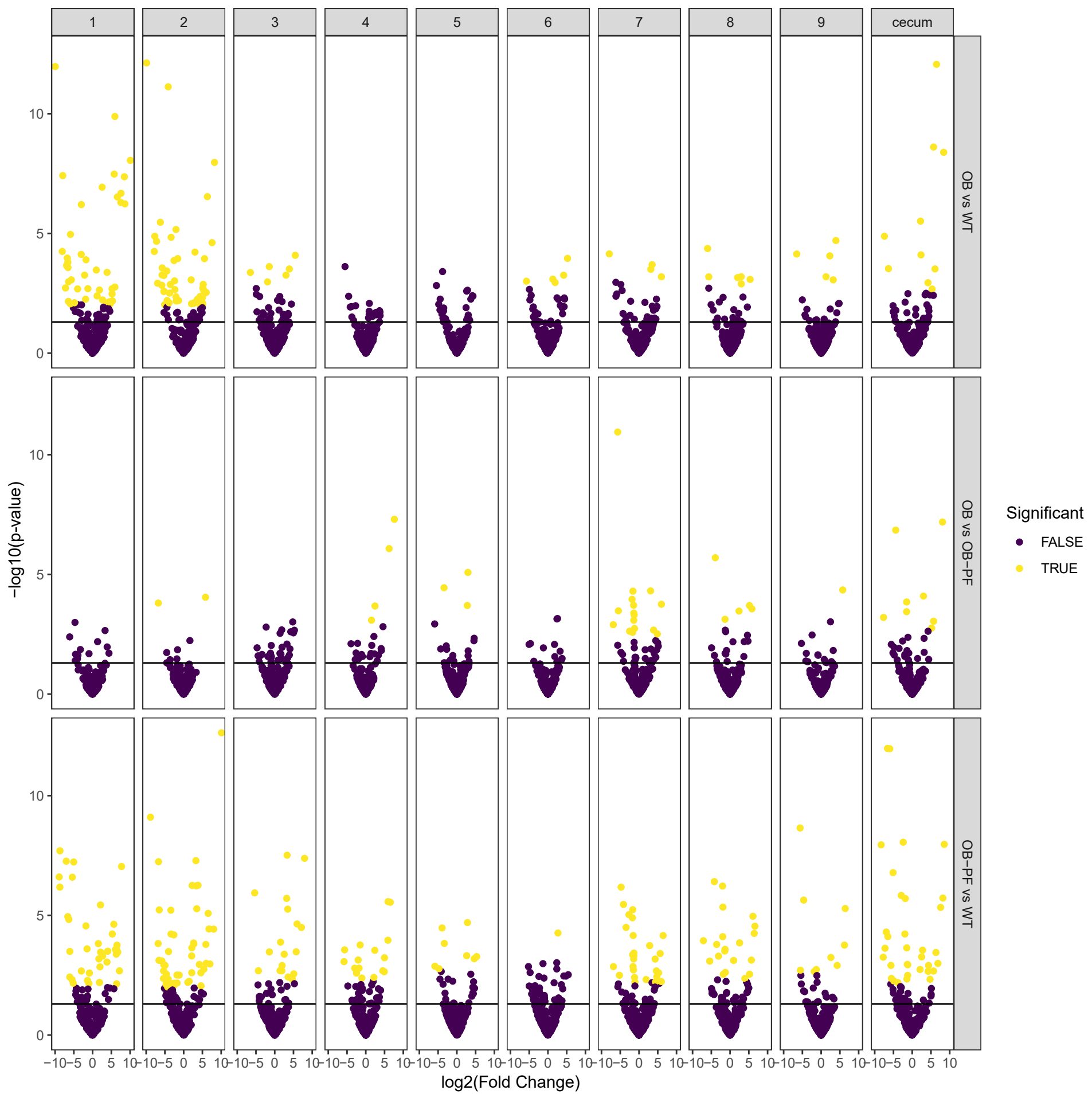

Supplement: Supplementary file 1 — Figure S1. Volcano plots separated by weeks showing gut microbial taxa that are differentially abundant in pairwise comparisons of wild-type mice fed ad libitum (WT), ob/ob mice fed ad libitum (OB) and ob/ob mice pair-fed according to WT intake (OB-PF). X-axis shows fold changes (in log2 scale) and Y-axis shows unadjusted p-values as reported by DESeq2 (in negative log10 scale). Each point represents an ASV, and its color represents whether a given ASV is significantly differentially abundant in that comparison after adjusting for multiple correction (as reported by DESeq2). (PDF 5436 kb) [file 42523_2019_7_MOESM1_ESM.pdf]

A

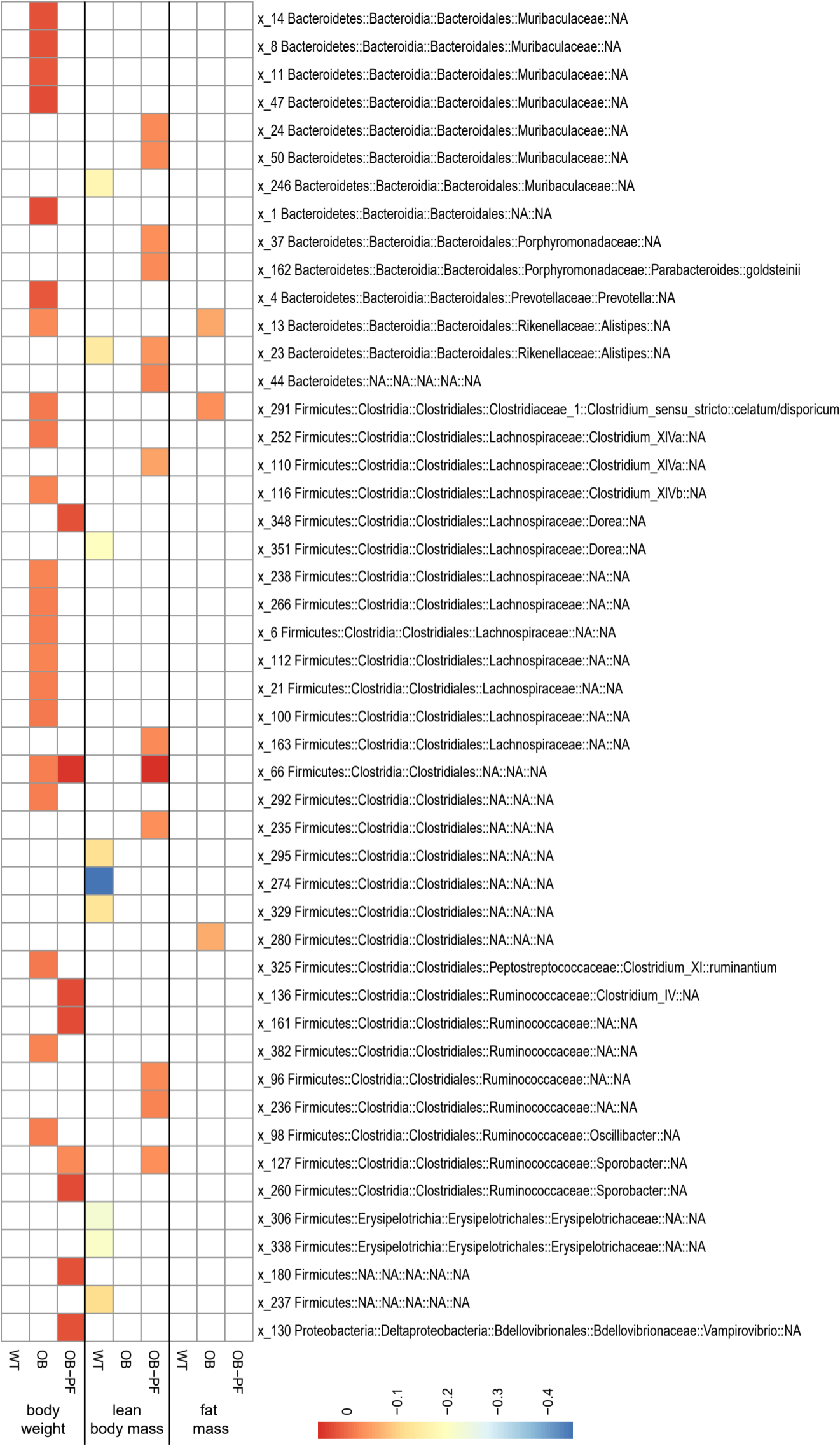

B

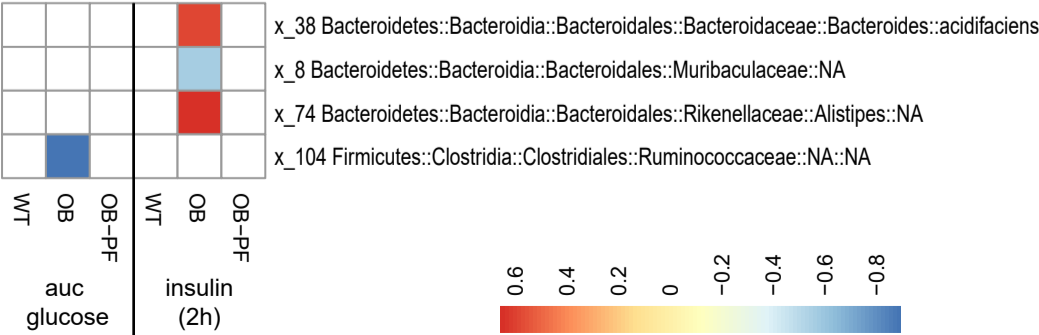

Supplement: Supplementary file 2 — Figure S2. Heatmaps showing associations between ASVs and host metabolic phenotypes within the three groups: wild-type mice fed ad libitum (WT), ob/ob mice fed ad libitum (OB) and ob/ob mice pair-fed according to WT intake (OB-PF). Associations that are not statistically significant are shown with white cells. (A) Effect size from linear mixed models between ASVs and three phenotypes: body weight, lean body mass and fat mass. (B) Spearman correlation co-efficient between ASVs and two phenotypes: area-under-the-curve for glucose response curves and insulin levels 2 h after glucose administration. (PDF 256 kb) [file 42523_2019_7_MOESM2_ESM.pdf]

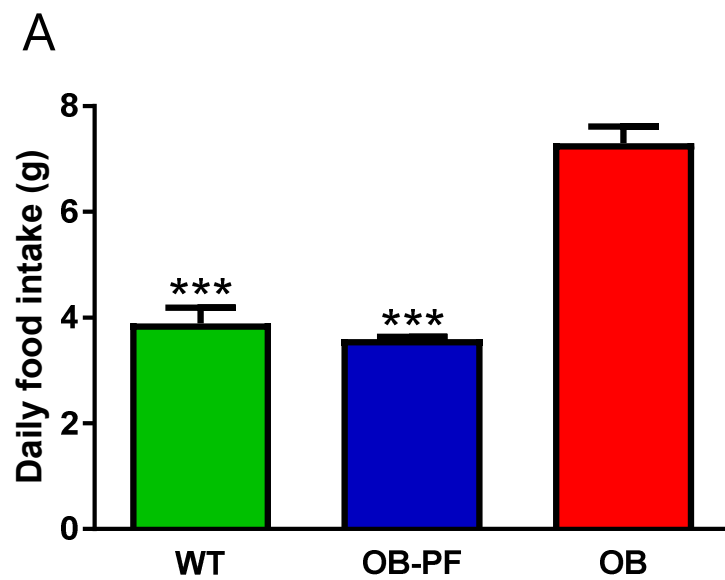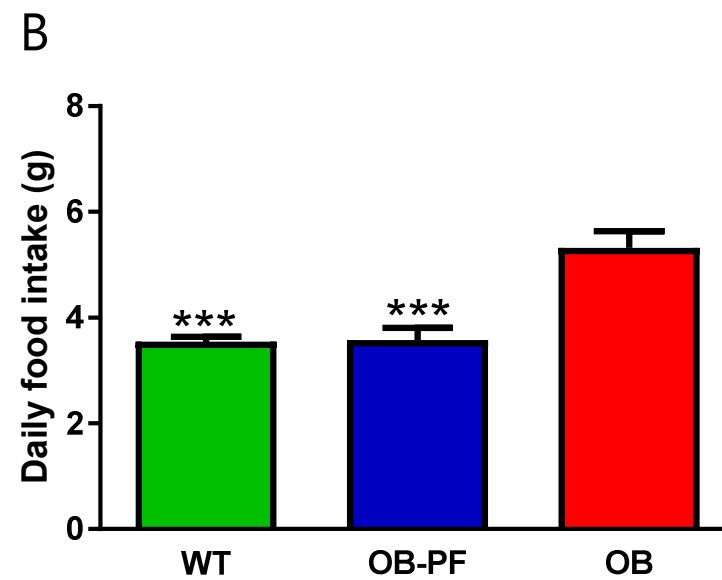

Supplement: Supplementary file 3 — Figure S3. Daily food intake of wild-type mice fed ad libitum (WT), ob/ob mice fed ad libitum (OB) and ob/ob mice pair-fed according to WT intake (OB-PF). Intake was precisely measured with TSE system (PhenoMaster, Bad Homburg, Germany) in week 3 (A) and week 9 (B). ***P < 0.001, for WT-vs-OB and OB-PF-vs-OB comparisons. (PDF 47 kb) [file 42523_2019_7_MOESM3_ESM.pdf]
